# Antimicrobial Resistance in the Era of Climate Change: Why We Should All Embrace and Integrate the One Health Approach in Clinical Practice?

**DOI:** 10.3390/antibiotics14101042

**Published:** 2025-10-17

**Authors:** Dimitris C. Kounatidis, Apostolos Evangelopoulos, Eleni V. Geladari, Angelos A. Evangelopoulos, Andreas Adamou, Sofia Kargioti, Charalampia V. Geladari, Maria Dalamaga, Vasileios Sevastianos, Natalia G. Vallianou

**Affiliations:** 1 Diabetes Center, Medical School, First Propaedeutic Department of Internal Medicine, Laiko General Hospital, National and Kapodistrian University of Athens, 11527 Athens, Greece; dimitriskounatidis82@outlook.com; 2 National and Kapodistrian University of Athens, 11527 Athens, Greece; apostolos.evangelopoulos.nak@gmail.com; 3 Third Department of Internal Medicine, Evangelismos General Hospital, 10676 Athens, Greece; elgeladari@gmail.com (E.V.G.); vsevastianos@gmail.com (V.S.); 4 Roche Diagnostics Hellas S.A., 15125 Athens, Greece; angelos.evangelopoulos@roche.com; 5 First Department of Internal Medicine, Sismanogleio General Hospital, 15126 Athens, Greece; adamou_andreas@yahoo.com (A.A.); skargioti@gmail.com (S.K.); 6 Hellenic Society of Environmental and Climate Medicine, 17455 Athens, Greece; gpath1@evaggelismos.gr; 7 Department of Biological Chemistry, Medical School, National and Kapodistrian University of Athens, 11527 Athens, Greece; madalamaga@med.uoa.gr

**Keywords:** antimicrobial resistance, climate change, global warming, gene transfer, One Health Approach

## Abstract

Antimicrobial resistance (AMR), recognized as one of the top ten global public health threats, is projected to cause around 10 million deaths annually by 2050. This trajectory can be averted by adopting the One Health Approach, which acknowledges the interconnection between human, animal, and environmental health. In this narrative review, we explore the multifactorial drivers of AMR, with particular emphasis on its relationship to climate change, examining the link between extreme weather events and the emergence of resistance. Furthermore, we highlight measures essential for mitigating both climate change and AMR. We provide a detailed account of the steps clinicians should implement in daily practice and underscore the importance of collaboration among individuals, healthcare professionals, livestock farmers, and agricultural workers to reduce AMR rates. Finally, we emphasize that interdisciplinary teams, organizations, and governments must work collectively within the concept of the One Health Approach to combat AMR.

## 1. Introduction

According to the World Health Organization (WHO), antimicrobial resistance (AMR) is among the ten greatest threats to global public health. AMR has been estimated to cause approximately 10 million deaths annually by 2050 [1,2]. This trajectory, however, could be prevented through the adoption of interventions such as access to clean water, sanitation and hygiene (WASH), along with other infection prevention and control strategies [3]. In hospital settings, healthcare-associated infections (HAIs) play a pivotal role in the emergence and spread of AMR [4].

The alarming rise in AMR has also been linked to climate change. Fossil fuels, including coal, oil, and gas, contribute to the greenhouse effect by releasing large amounts of carbon dioxide, ozone, nitric oxide, and methane into the atmosphere [5]. Human activities such as industrial production, transportation, urbanization, deforestation, and agriculture drive these greenhouse gas (GHG) emissions. GHG emissions are related to global temperature increases and extreme weather events such as floods, droughts, and wildfires [5,6,7]. These environmental changes can facilitate the emergence and transmission of resistant pathogens.

The One Health Approach, which recognizes the intricate interplay between human, animal, and environmental health, provides a framework for addressing AMR [7,8]. In this narrative review, we explore the multifactorial drivers of AMR, highlighting the role of climate change and emphasizing the necessity of implementing One Health Approach strategies in clinical practice and public health policies.

## 2. Antimicrobial Resistance

### 2.1. How Does AMR Develop?

AMR refers to the ability of microorganisms to withstand the effects of currently available antibiotics, antivirals, antifungals, and antiparasitic agents. Among these, antibiotic resistance represents the most significant concern.

**Figure 1:**
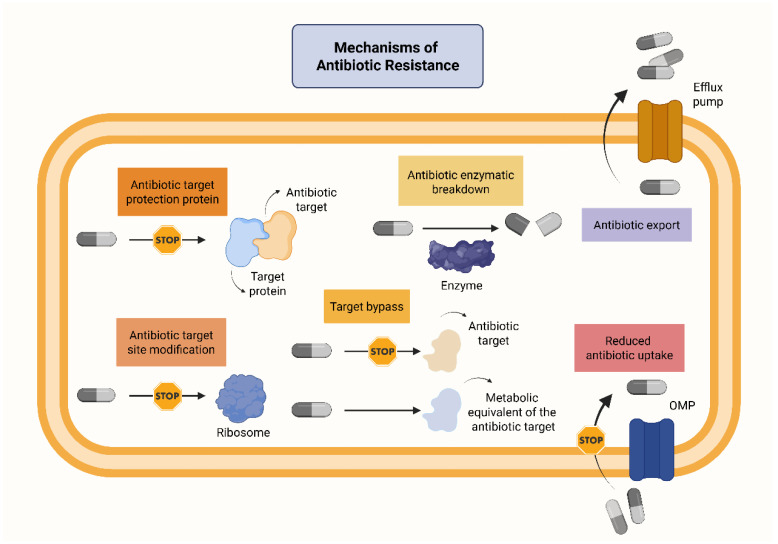
Main mechanisms of antibiotic resistance. The four major mechanisms of AMR are: (i) modification of antibiotic target sites, (ii) inactivation of antibiotics by specific enzymes, (iii) active efflux of antibiotics via efflux pumps, and (iv) reduced antibiotic penetration into bacterial cells, particularly through loss of porins in the outer membrane of Gram-negative bacteria. Abbreviations: OMP: Outer membrane protein. Created in BioRender. Kounatidis, D. (2025) https://BioRender.com/cv5fkpy. Assessed on 13 September 2025.

The molecular mechanisms underlying antibiotic resistance include: (i) Modification of antibiotic target sites: This mechanism is exemplified by methicillin-resistant *Staphylococcus aureus* (MRSA) and *Vancomycin-Resistant enterococci* (VRE). In MRSA, acquisition of the *mecA* gene leads to the production of penicillin-binding protein PBP2a, which exhibits reduced affinity for methicillin. Consequently, methicillin binding is impaired or entirely inhibited. In VRE, acquisition of *Van* genes induces structural changes in the peptidoglycan layer of Gram-positive bacteria, reducing or eliminating vancomycin binding [9]. (ii) Inactivation or alteration of antibiotics: A well-known example is β-lactamases, enzymes that hydrolyze the β-lactam ring of antibiotics, rendering them ineffective. Carbapenemases, a type of β-lactamases, currently pose a major threat in hospital settings. Antibiotic alterations can also occur through chemical modifications, such as the addition of adenyl, phosphoryl, or acetyl groups, which are typical in aminoglycoside resistance. These modifications prevent aminoglycosides from exerting their antibacterial effect. (iii) Activation of efflux pumps: Efflux pumps actively transport antibiotics out of bacterial cells, reducing intracellular drug concentrations. Efflux pumps may confer resistance to multiple antibiotic classes, turning bacterial strains into multidrug-resistant organisms (MDROs). (iv) Reduced penetration of antibiotics: resistance can also arise from decreased membrane permeability. In Gram-negative bacteria, alterations in outer membrane proteins (OMPs), particularly porins, can limit antibiotic entry. Porins are water-filled channels that allow passive diffusion of mainly hydrophilic substances. Mutations in porin-encoding genes can lead to porin loss, thereby reducing intracellular antibiotic levels and contributing to resistance [9,10,11,12,13,14,15]. Figure 1 illustrates the major mechanisms by which bacterial cells develop AMR.

It is important to note that most resistance genes are not recent innovations. They are often mobilized from environmental bacteria that naturally harbor them as a defensive mechanism. These genes may protect bacteria against antibiotics produced by themselves or other microorganisms. Additionally, a distinction exists between microbial resistance arising from spontaneous mutations, such as modifications to target sites or porins, and resistance acquired via horizontal gene transfer (HGT), which typically involves genes encoding inactivating enzymes or highly efficient efflux pumps. In this context, the role of climate change in the development of AMR is particularly significant. Rising temperatures have been linked to increased prevalence of several pathogenic bacteria responsible for foodborne diseases, including *Salmonella* spp. and *Listeria monocytogenes*. Extreme weather events, such as hurricanes and flooding, are associated with heightened risks of waterborne and foodborne infections. During droughts in Africa, for example, a 5% increase in the incidence of diarrheal episodes among children has been documented [16]. Moreover, transmission of antibiotic resistance genes (ARGs) is more likely during extreme weather events, as contaminated water can more readily enter the food supply [14,15,16].

### 2.2. The Role of the Resistome and Biofilm Formation

AMR encompasses the entire pool of ARGs, collectively referred to as the “resistome.” This pool includes vertically transferred (VT) genes, which confer intrinsic resistance (IR) within the same taxa or taxa-specific genes; horizontally transferred (HT) genes, which confer resistance across different taxa or non-specific taxa; and silent genes, which have the potential to confer resistance, but are not actively expressed [9,15]. Among the major pathogens exhibiting IR worldwide is the ESKAPE group, which includes *Enterococcus faecium*, *Staphylococcus aureus*, *Klebsiella pneumoniae*, *Acinetobacter baumannii*, *Pseudomonas aeruginosa*, and *Enterobacter* species. In addition to intrinsic resistance, these pathogens can acquire resistance via HT genes and are known to frequently form biofilms [9,15].

**Figure 2:**
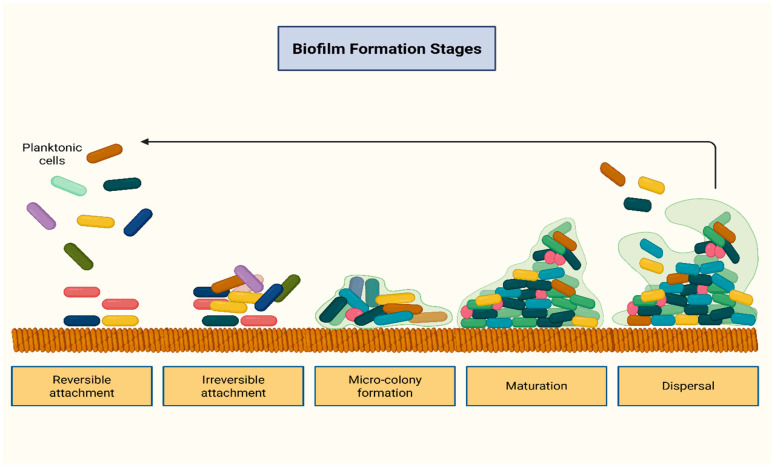
Stages of biofilm formation. Biofilm formation begins with bacterial attachment to a surface, followed by accumulation into micro-colonies. Within these micro-colonies, bacteria aggregate and mature into larger colonies. During this process, quorum sensing systems interact with transcriptional factors, coordinating bacterial behavior and promoting biofilm persistence and development. Created in BioRender. Kounatidis, D. (2025) https://BioRender.com/68flolu. Assessed on 5 October 2025.

Biofilms are structured communities of microorganisms that may originate from a single or multiple bacterial strains and typically adhere to surfaces. They serve as a survival strategy, allowing bacteria to aggregate, protect themselves from other microorganisms, endure extreme conditions such as nutrient and oxygen deprivation, and facilitate dispersal. Nearly all bacterial species are capable of forming biofilms [9,15]. Biofilm formation occurs through a sequence of stages, including initial attachment, bacterial accumulation and maturation, followed by detachment and dispersal. Intercellular communication, mediated by the quorum sensing system (QSS), is essential for biofilm development. The QSS coordinates bacterial behavior through the release of signaling molecules, while the production of the extracellular polymeric substances (EPS) matrix stabilizes the biofilm structure. Figure 2 illustrates the stepwise process of biofilm formation and development.

Within a biofilm, bacterial cells in different layers exhibit distinct growth and metabolic rates, which influence their responses to antibiotic exposure and contribute to resistance development. The EPS matrix further hinders antibiotic penetration, predisposing biofilm-associated bacteria to resistance. Notably, within this community, there are resistant cells known as “persisters”. These cells adopt a spore-like state allowing survival under oxygen or nutrient limitation and local pH fluctuations. Although non-dividing, persisters enhance the overall survival of the community, as they can resume activity once detached and dispersed. The close intercellular adherence and dense EPS matrix also facilitate HGT, as high cell density and proximity create ideal conditions for the exchange of resistance genes. Therefore, biofilm formation, while enabling microbial communication, remains central to the persistence and spread of AMR [9,10,11,12,13,14,15,16].

**Figure 3:**
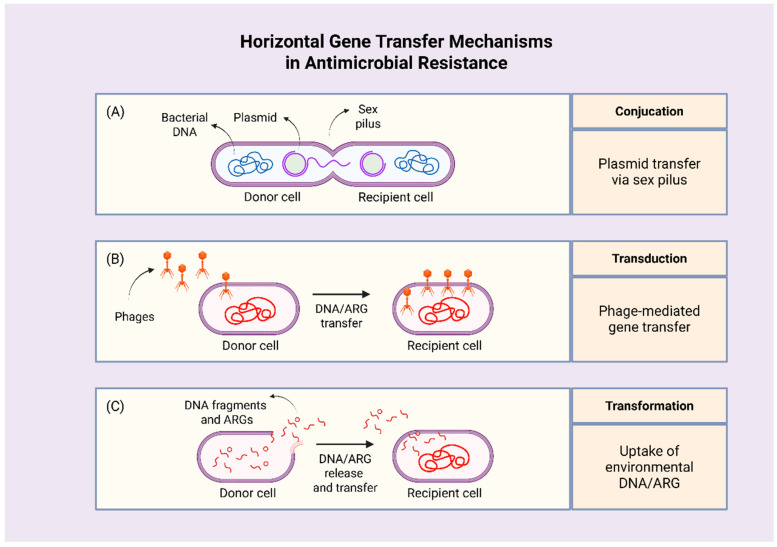
Mechanisms of horizontal gene transfer driving antimicrobial resistance. Antimicrobial resistance is spread among bacteria through three main mechanisms: (**A**) conjugation, in which genetic material is transferred directly between cells or via a sex pilus; (**B**) transduction, mediated by bacteriophages that deliver donor genes into recipient cells; (**C**) and transformation, where bacteria uptake foreign DNA from the environment, acquiring new traits, such as antibiotic resistance genes. Abbreviations: ARGs: antimicrobial resistance genes. Created in BioRender. Kounatidis, D. (2025) https://BioRender.com/0ksp2l5. Assessed on 12 September 2025.

Bacteria can also acquire resistance through conjugation, transformation, and transduction. Conjugation involves the transfer of genetic material from a donor to a recipient cell, either via direct contact or through a sex pilus. Transduction is mediated by bacteriophages, which invade bacterial cells and integrate donor genes, including ARGs, into the recipient genome. Lastly, transformation occurs when a bacterial cell uptakes foreign DNA from its environment, acquiring novel traits such as ARGs; this process can also be applied in laboratory settings to produce genetically engineered microorganisms [9,16,17]. Figure 3 illustrates the major HGT mechanisms that facilitate the spread of ARGs among bacteria.

HT genes, like plasmids—extrachromosomal DNA capable of independent replication—and mobile genetic elements (MGEs), such as transposons, integrons, and insertion sequences, play a vital role in transferring resistance genes across species and genera. A notable example is the transfer of the vancomycin-resistance gene *VanA* from *Enterococcus* to *Staphylococcus aureus* [9,12,18].

### 2.3. Drivers of AMR

AMR is a multifactorial phenomenon driven by the widespread use of antimicrobials in humans and animals, extensive application of disinfectants and antiseptics, economic factors, the food chain and food industry, and climate change. These drivers collectively accelerate the emergence and spread of resistance [19,20,21].

Disinfectants, which contain one or more antimicrobial compounds such as chlorine, chlorhexidine, iodine, alcohols, ammonium, silver, triclosan, and hydrogen peroxide, are essential for preventing infections in both humans and animals, maintaining hygiene in the food industry, and ensuring safe water supplies. However, similar to antibiotics, disinfectants are frequently overused or misused, with usage increasing exponentially since the coronavirus disease 2019 (COVID-19) pandemic. Sub-optimal concentrations, improper application, or insufficient contact time can compromise their effectiveness, allowing bacteria to survive and spread, thereby contributing to resistance development [19,20,21,22]. For example, inadequate hand hygiene practices or architectural barriers, such as angular hospital surfaces, may reduce disinfectant efficacy, leaving microbial populations exposed to insufficient doses.

The role of disinfectants in rising AMR rates must not be overlooked. Their use should follow international guidelines, and be limited to cases where clearly necessary, including healthcare or aesthetic procedures, to minimize overuse and the risk of resistance. Notably, bacteria can develop resistance to disinfectants alongside antibiotics, a phenomenon known as “co-resistance.” [22]. Chen et al. demonstrated that exposure of *Klebsiella pneumoniae* to NaClO led to co-resistance not only to the disinfectant but also to antibiotics such as colistin, ciprofloxacin, tetracycline, gentamicin, and erythromycin [23]. Similarly, Wang et al. reported “co-resistance” among wastewater bacteria exposed to quaternary ammonium compounds, showing resistance to colistin, ciprofloxacin, kanamycin, azithromycin, and ampicillin [24]. During the COVID-19 pandemic, quaternary ammonium compounds and trihalomethanes in water and soil near Wuhan hospitals were linked to the emergence of ARGs, including *qepA*, encoding a quinolone efflux pump, and *oxa-20*, encoding β-lactamase resistance [25].

Antiseptics, unlike disinfectants, are applied only to living tissues and are widely used in surgical and healthcare settings. Common examples include alcohol, iodine (typically as povidone-iodine), hydrogen peroxide, and triclosan. As with disinfectants, inappropriate or excessive use of antiseptics can contribute to AMR [17,18,19,20,21]. Overall, disinfectants and antiseptics represent a cryptogenic but significant source of AMR. Their widespread and sometimes inappropriate application, particularly intensified during the COVID-19 pandemic, underscores the need for stricter regulation and rational use.

#### 2.3.1. Healthcare-Associated Factors and AMR

Alongside hidden drivers of AMR, such as inadequate antimicrobial policies, weak surveillance systems, antimicrobial use in agriculture, and climate change, healthcare-associated factors play a central role. Table 1 presents the major drivers of AMR.

Hospitals remain primary settings where AMR emerges and spreads. While antibiotic administration is often life-saving, overuse and misuse have been linked to resistance development. A cross-sectional survey across five hospitals in London revealed gaps in antimicrobial prescribing knowledge among junior doctors, with 74% reporting that they should have received more education on appropriate prescribing practices [26]. These findings underscore the need for continuous medical education, not only for early-career physicians but across the entire healthcare workforce, to ensure prudent antibiotic use. Several hospital-specific factors further drive AMR, including patients’ comorbidities, prolonged hospital stays, and the widespread use of invasive medical devices. Intensive care units (ICUs), where patients frequently require intubation and broad-spectrum antibiotics, represent high-risk environments for MDROs, and neonatal ICUs (NICUs) face similar challenges. Robust infection control and prevention (ICP) strategies are therefore essential to reduce the burden of HAIs and AMR in these vulnerable settings [27,28].

Demographic changes also contribute significantly. According to the United Nations Population Division, individuals aged >65 years numbered 770 million in 2022 and are projected to reach approximately 1.5 billion by 2050 [29]. Aging, compounded by immunosenescence and the rising prevalence of comorbidities such as cancer and diabetes, increases susceptibility to infection and fosters AMR [9,30]. Similarly, the global increase in immunocompromised patients represents an expanding reservoir for MDROs. Beyond hospitals, antibiotic misuse in the community is a major concern. In primary care, inappropriate prescribing rates have been estimated at 88% in Pakistan and approximately 28% in the United States between 2010 and 2015 [30,31]. Notably, in the US, 80–90% of all antibiotic prescriptions are issued in outpatient settings [32]. These data highlight the substantial role of community-level prescribing practices in shaping the trajectory of AMR.

#### 2.3.2. Animal Farming, Food Production, and AMR

The exponential growth of the global human population has driven extensive animal farming. In Asia, for example, daily animal protein intake has more than tripled over the past fifty years [33,34]. Rising demand for animal products has been closely linked to increasing rates of AMR, with an estimated 50–80% of the tens of thousands of tons of antibiotics used annually consumed in animal farming [35]. This reflects not only their use for food safety but also their frequent application as growth promoters. Sub-therapeutic antibiotic doses in livestock reduce morbidity and mortality while increasing production yields [36]. To mitigate the AMR risk, Sweden became the first country to ban antibiotics as growth promoters in 1986, followed by the European Union in 2006, the United States in 2015, and China in 2020 [37,38,39,40]. However, several countries, particularly low- and lower-middle-income nations such as Brazil, India, and South Africa, have yet to adopt these restrictions [37].

Alternative strategies are being explored to reduce antibiotic reliance in animal farming. Probiotics (live beneficial microorganisms) and prebiotics (fibers) have been shown to improve gut microbiota diversity and composition, conferring survival advantages to animals and reducing the need for antibiotics. Vaccination in animals has also demonstrated benefits, promoting animal health while minimizing antibiotic misuse [38,39,40].

AMR originating in animals has already spread to humans, both directly through food supply chains and indirectly via microbial alterations in ecosystems [41]. Antibiotic residues from animal production interact with environmental bacteria, driving broader AMR. Colistin illustrates this risk. First introduced in 1947, it was widely used in poultry farming, and in 2013 the plasmid-mediated colistin resistance gene *mcr-1* was identified in *Escherichia coli* from pigs in China [50]. Since then, *mcr-1* has spread globally among humans, poultry, and pigs, threatening the efficacy of colistin—the “last-resort” antibiotic [51,52]. Extended-spectrum β-lactamases (ESBLs) and carbapenem resistance in ESKAPE pathogens have also been linked to inappropriate antibiotic use in animal agriculture [52,53].

Transmission of AMR from animals to humans occurs both directly and indirectly. Direct transmission may occur through the consumption of contaminated meat, dairy, or eggs. For instance, rising fluoroquinolone resistance in *Campylobacter* spp. from chickens parallels resistance observed in human cases of gastroenteritis [53]. On the other hand, indirect transmission results from waste generated by intensive animal farming, which introduces large amounts of ARGs into the environment. ARG concentrations in manured soil have been estimated to be 28,000 times higher than in non-manured soil [54]. Studies have also detected ARGs in crops grown in manured soil, as well as in raw meat, fish, vegetables, and fruits, highlighting the interconnectedness of environmental, plant, and food reservoirs in the spread of AMR [55,56].

Over 70% of antibiotics administered to animals are excreted unchanged or as active metabolites into the environment, contaminating water and soil and disrupting aquatic, plant, and soil microbiomes. These resistance traits can then be transmitted to humans through the food chain [57,58,59]. As mentioned above, antibiotics are frequently released into the environment through manure. When this manure is applied as fertilizer, it provides a direct pathway for ARGs to enter the soil and crops, ultimately allowing their transmission to humans through the food chain. Aquaculture is an emerging contributor to AMR. With production expected to double by 2030, it exacerbates global warming and enhances the dissemination of ARGs [57,58,59]. AMR development along the food chain can be halted through the One Health Approach.

**Figure 4:**
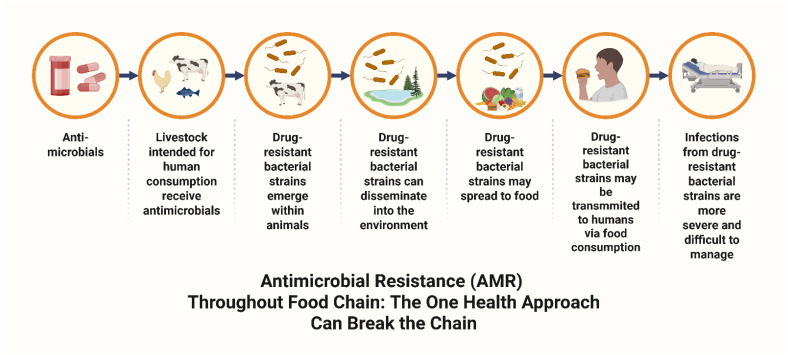
Antimicrobial resistance development along the food chain. This figure depicts the progression of AMR along the food chain, from antimicrobial use in food-producing animals to the emergence of drug-resistant bacteria and their potential transfer to the environment and humans. The adoption of the One Health Approach is vital in mitigating AMR. Abbreviations: AMR: antimicrobial resistance. Created in BioRender. Kounatidis, D. (2025) https://BioRender.com/zt7667r. Assessed on 5 October 2025.

The One Health Approach provides a holistic framework to address AMR by integrating human, animal, plant, and environmental health. This perspective recognizes that human well-being is interconnected with animal and environmental health. In the Anthropocene, factors such as climate change, global travel and trade, extensive animal farming, deforestation, and urbanization contribute to the spread of zoonotic diseases and AMR. Diseases such as West Nile virus, rabies, *Salmonella*, Lyme disease, Q fever, and brucellosis have spread worldwide, highlighting the global importance of coordinated action. The One Health Approach aims to mitigate both zoonotic diseases and AMR through integrated national and international policies, legislation, and collaborative interventions across sectors [57,58,59]. Figure 4 illustrates the development of AMR along the food chain, emphasizing that adopting the One Health Approach is crucial to breaking this chain.

#### 2.3.3. Economical Components as Drivers of Antimicrobial Resistance: Is Geographical Distribution Implicated in Antimicrobial Resistance?

Currently, sub-Saharan Africa and South Asia bear the highest burden of AMR. According to the Global Burden of Disease Collaborators on AMR (2021), South Asia, Latin America, and the Caribbean are projected to experience the highest all-age AMR-associated mortality rates by 2050. These rates are expected to rise particularly among individuals aged over 70 years. Notably, many low- and lower-middle-income countries lack antimicrobial stewardship policies and antibiotic surveillance systems. Inadequate hospital infrastructure, such as overcrowded wards, limited isolation capacity, and insufficient infection prevention measures, further facilitates the spread of AMR within healthcare settings. Additionally, the vulnerability of populations in low-income countries, driven by limited access to clean WASH, contributes to a higher incidence of infectious diseases, increased antibiotic use, and consequently, the development of AMR. Collectively, social, economic, and policy-related factors intersect to shape the global public health challenge of antimicrobial resistance [3].

## 3. Climate Change as a Driver of Antimicrobial Resistance: Data and Trends

Climate change has been increasingly associated with extreme weather events, including rising ambient temperatures, flooding, droughts, and pollution. Variations in temperature, pH, oxygen levels, and nutrient availability may promote the proliferation of resistant strains capable of surviving under harsh environmental conditions. These environmental shifts alter microbial community composition and functionality, often favoring the dominance of resistant microorganisms [57,58,59].

Warming climates, in particular, facilitate the survival and spread of pathogenic bacteria in aquatic ecosystems. For instance, *Vibrio parahaemolyticus*, a Gram-negative bacterium that thrives in warm marine environments, exemplifies this trend. It causes gastroenteritis following the consumption of contaminated seafood, particularly shellfish [59,60]. In the United States, species of the *Vibrio* genus are the leading cause of seafood-borne gastroenteritis outbreaks, with *V. parahaemolyticus* responsible for the majority of cases [59,60]. Beyond foodborne illness, this pathogen can also cause wound infections after exposure to contaminated water and, in severe instances, septicemia [59,60]. Mahhiedine et al. investigated 20 clinical isolates of *V. parahaemolyticus* collected in Quebec, between 2018 and 2022, demonstrating that growth, motility, biofilm formation, hemolysin production, and AMR, particularly against β-lactams, kanamycin, and streptomycin, were temperature-dependent. Increasing temperatures enhanced virulence by upregulating hemolysin expression and altering QSS involved in biofilm development, resulting in greater adherence and biofilm formation [61].

Another clinically significant species, *Vibrio cholerae*, causes cholera outbreaks via ingestion of contaminated water. *Vibrio cholerae* proliferates more rapidly in warm aquatic environments, especially in tropical and subtropical regions [62]. Imoli et al. examined *V. cholerae* O1 strains isolated during a 2022 cholera outbreak in Kenya and reported that 99% of isolates were MDROs, 99% resistant to azithromycin, and 98.5% were ESBL producers [63]. Additionally, El-Liethy et al. linked increasing temperatures and heavy rainfall to cholera outbreaks across Africa, identifying *V. cholerae* O1 and O39 strains as key contributors [64]. Notably, between 2000 and 2023, Africa reported an estimated 2,727,172 cholera cases, underscoring the severe AMR burden [65]. On the other hand, in China, Li et al. documented rising azithromycin resistance in *V. cholerae* O39 strains isolated in Anhui Province between 2013 and 2024 [66].

Melting glaciers in polar regions represent another potential climate change-driven source of AMR. Ancient resistance genes preserved in ice have recently been characterized. Thajudeen et al. identified 154 ARGs in Arctic and Antarctic glaciers conferring resistance to fosfomycin, vancomycin, tetracycline, β-lactams, and bacitracin [67]. These findings suggest that glacier melting could contribute to the environmental resistome, potentially reintroducing ancient ARGs into modern microbial populations [67,68]. ARGs predate human antibiotic use. Indeed, resistance to β-lactams is estimated to have emerged roughly 2 billion years ago, and vancomycin resistance about 0.5 billion years ago [68]. Advanced molecular analyses in polar regions could therefore expand our understanding of the evolutionary origins of the environmental resistome.

Beyond aquatic ecosystems, rising ambient temperatures have been correlated with increasing AMR in other bacterial species. MacFadden et al., analyzing data across the United States, found that every 10 °C rise in temperature was associated with a 5.1% increase in AMR in *E. coli*, a 3.5% increase in *Klebsiella pneumoniae*, and a 3.1% increase in *Staphylococcus aureus* [69]. Similarly, Kaba et al. reported that carbapenem resistance in *Pseudomonas aeruginosa*, *K. pneumoniae*, *E. coli*, and MRSA was positively correlated with increasing temperatures across 30 European countries. Their projections indicated that by 2039, carbapenem-resistant *P. aeruginosa* prevalence may double in some northern and western European regions [70].

Bloomfield et al. analyzed community-associated MRSA (CA-MRSA) infections in Northern Western Australia (2004–2018). Among 57,557 cases, strains carrying *Panton-Valentine leukocidin* (PVL) genes rose from 3.4% to 59.8%. They found that high temperature and humidity favored the spread of PVL-positive CA-MRSA, particularly the WA121 clone [71]. This clone carries MGEs, including the methicillin-resistance cassette SCCmecIVo and transposon Tn4791, which have also been horizontally transferred to *Staphylococcus argenteus* [72].

Foodborne diseases likewise exhibit climate-driven seasonality. *Salmonella* infections peak during summer across Europe and are more frequent in southern than northern regions, reflecting the enhanced growth of *Salmonella* spp. at higher temperatures [73]. Typhoid fever poses an additional burden in low- and lower-middle-income countries, where inadequate food processing and hygiene practices exacerbate infection rates and economic costs [74,75]. Elevated temperature and humidity further compromise food safety by affecting food preservation, while poor sanitation, unsafe water, and improper handling amplify the risk of infection [76,77,78,79,80,81,82,83,84,85,86].

**Figure 5:**
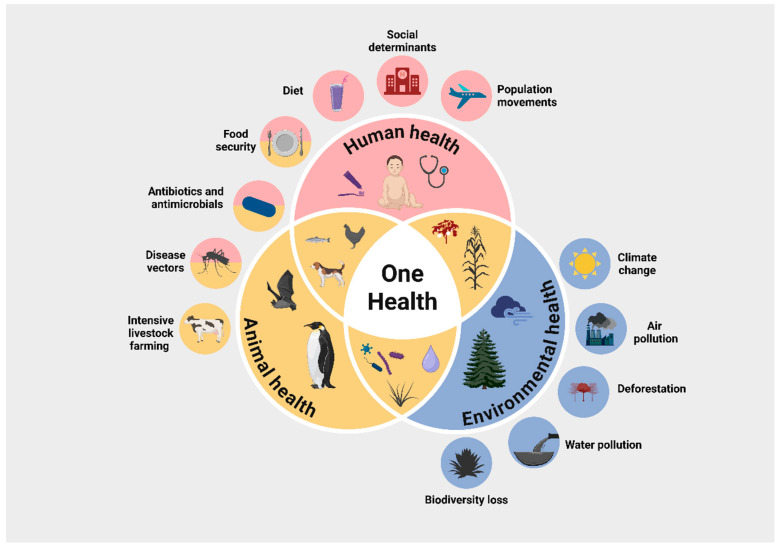
The One Health Approach. This figure demonstrates the interdependence between human, animal, and environmental health. It shows how global challenges, including climate change, rising temperatures, air pollution, glacier melting, intensive livestock production, deforestation, biodiversity loss, increased mobility and trade, food insecurity, and inappropriate antibiotic use, contribute to the emergence and spread of AMR. Created in BioRender. Kounatidis, D. (2025) https://BioRender.com/tsk5a7d. Assessed on 5 October 2025.

Air pollution has also emerged as a significant contributor to AMR. The atmosphere itself acts as a reservoir for ARGs [87,88,89]. Gao et al. [87] analyzed 392 atmospheric metagenomic samples from Asia, Europe, America, and Oceania, identifying 1863 ARGs primarily conferring resistance to tetracyclines, macrolides, lincosamides, streptogramins, and multidrug resistance. Many samples were also contaminated with chemical pollutants and particulate matter [87]. Zhou et al. [89], in a global study analyzing over 11.5 million samples from 116 countries (2000–2018), found a direct relationship between fine particulate matter (PM_2.5_) and AMR. A 1% rise in PM_2.5_ concentration corresponded to a 0.5–1.9% increase in AMR prevalence. They further estimated that achieving the WHO-recommended PM_2.5_ target of 5 µg/m^3^ by 2050 could reduce global AMR levels by 16.8% [88,89]. Figure 5 illustrates the One Health Approach, highlighting the close interconnection between human, animal, and environmental health, as well as the global factors contributing to the emergence and spread of AMR.

## 4. Challenges and Future Perspectives in Clinical Management

Several factors contribute to the emergence and persistence of AMR in hospital settings. These include the administration of suboptimal antibiotic doses, prolonged antimicrobial therapy, extended hospital stays, failure to implement the de-escalation approach once microbiological data become available, and the overall overuse of antibiotics. Before initiating antibiotic therapy, clinicians must carefully evaluate whether such treatment is truly warranted. When indicated, patients should receive the appropriate antimicrobial agent, at the correct dose and for an optimal duration, based on thorough clinical assessment and laboratory evidence. Microbiological laboratories play a key role in combating AMR, and access to well-equipped facilities utilizing advanced molecular diagnostic tools, such as polymerase chain reaction (PCR), next-generation sequencing (NGS), and Clustered Regularly Interspaced Short Palindromic Repeats/CRISPR-associated protein 9 (CRISPR/Cas9) gene-editing technologies, is essential. These technologies allow for rapid and precise identification of pathogens and their resistance determinants, enabling more targeted therapeutic decisions [90,91,92,93,94].

The implementation of the “step-down” or de-escalation strategy, which involves reassessing antimicrobial therapy every 48 h and modifying it according to microbiological results, has proven effective in reducing AMR. Furthermore, antimicrobial stewardship programs (ASPs) and infection control and prevention (ICP) measures should be firmly embedded in routine clinical practice [94,95,96,97]. Equally important is adherence to the WASH strategy. Regular training sessions on proper hand hygiene for all healthcare workers must be standardized across healthcare facilities. Given the continuous influx of new personnel, including residents and nurses, ongoing education, evaluation, and feedback on handwashing practices are indispensable for maintaining compliance and minimizing transmission risks [94,95,96,97].

Beyond clinical and hospital-based interventions, addressing climate change is imperative, as it represents an emerging and significant driver of AMR. Mitigation efforts require coordinated action at both individual and global levels, through collaboration among multidisciplinary teams across human, animal, and environmental health sectors. Achieving these goals demands not only behavioral and institutional commitment but also the enactment of robust policies and legislation regulating animal farming, deforestation, urban green space preservation, and GHG emission reduction, all within the broader framework of public and planetary health [98,99,100,101,102].

## 5. Conclusions

AMR remains one of the most substantial global public health threats, demanding coordinated and sustained action. Clinicians must take a leading role in embracing and promoting the One Health Approach, which recognizes the interconnectedness of human, animal, and environmental health. As discussed, AMR is a multifactorial and dynamic phenomenon that requires comprehensive, evidence-based strategies to mitigate its impact. Effective collaboration among individuals, healthcare professionals, veterinarians, agricultural workers, policymakers, organizations, and governments is crucial to curbing its spread. Moreover, the intricate and not yet fully elucidated relationship between climate change and AMR further highlights the urgency of a unified response. This interconnection reinforces the necessity for multidisciplinary teams and coordinated national and international initiatives operating within the framework of the One Health Approach. Only through collective and integrated action can we hope to slow the progression of AMR and safeguard public health for future generations.

## Figures and Tables

**Table 1:** Major drivers of antimicrobial resistance.

Drivers of AMR	Setting/Paradigm
Inappropriate use of disinfectants [17,18,19,20,21]	Healthcare-associated or in the community
Inappropriate use of antiseptics [17,18,19,20,21]	Healthcare-associated or in the community
Overuse and misuse of antibiotics in healthcare settings [22,23,24,25,26,27,28]	Hospitals or other healthcare-associated settings (i.e., nursing homes)
Aging population [29,30]	More prone to epigenetic modifications and AMR
Overuse and misuse of antibiotics in the community [31,32]	By primary care physicians in the community
Overuse and misuse of antibiotics for the growth of animals by farmers [33,34,35]	Animal sector
Misuse of antibiotics in agriculture [36]	Plant workers
Lack of Legislation [37,38,39,40]	Policies, such as those regarding farming, growing crops and antibiotics surveillance
Socioeconomic factors [41]	Poor sanitation in low-income countries
Inappropriate handling of food [42,43]	Food supplies in low-income countries
Inappropriate handling of sewages [44,45,46]	From hospitals, pharmaceutical companies, industries
Climate change [18,46,47,48,49]	Increasing temperatures, humidity, heavy rainfalls, flooding, droughts

Abbreviations: AMR: antimicrobial resistance.

## Data Availability

No new data were created or analyzed in this study. Data sharing is not applicable to this article.

## Abbreviations

AMR: antimicrobial resistance; ARGs: antibiotic resistant genes; CA-MRSA: community-associated methicillin-resistant Staphylococcus aureus; CRISPR/Cas9: clustered regularly interspaced palindromic repeats; EPS: extracellular polymeric substances; ESBL: extended spectrum beta lactamases; GHGs: greenhouse gasses; HAI: healthcare-associated infections; HT: horizontal transfer; ICP: infections control and prevention; ICU: intensive care unit; IG: integron; IR: intrinsic resistance; MDROs: multi-drug resistant microorganisms; MGEs: mobile genetic elements; NGS: next-generation sequencing; NICU: neonatal intensive care unit; OMPs: outer membrane proteins; PCR: polymerase chain reaction; PVL: Panton Valentine leucocidin; QSS: quorum sensing system; Tn: transposon; VT: vertical transfer; WASH: water supplementation, sanitation and hygiene; WHO: World Health Organization.
